# Transdermal Delivery of Cannabidiol for the Management of Acute Inflammatory Pain: A Comprehensive Review of the Literature

**DOI:** 10.3390/ijms25115858

**Published:** 2024-05-28

**Authors:** Ève Lefebvre, Nancy Tawil, L’Hocine Yahia

**Affiliations:** 1 Department of Mechanical Engineering, Polytechnique Montréal, Montréal, QC H3T 1J4, Canada; lhocine.yahia@polymtl.ca; 2 Qeen BioTechnologies, Gatineau, QC J9J 3K3, Canada; tawil@qeen-bio.com

**Keywords:** cannabidiol, transdermal, delivery, polymeric, inflammation, arthritis, cannabis

## Abstract

The emerging field of nanotechnology has paved the way for revolutionary advancements in drug delivery systems, with nanosystems emerging as a promising avenue for enhancing the therapeutic potential and the stability of various bioactive compounds. Among these, cannabidiol (CBD), the non-psychotropic compound of the *Cannabis sativa* plant, has gained attention for its therapeutic properties. Consequently, researchers have devoted significant efforts to unlock the full potential of CBD’s clinical benefits, where various nanosystems and excipients have emerged to overcome challenges associated with its bioavailability, stability, and controlled release for its transdermal application. Therefore, this comprehensive review aims to explain CBD’s role in managing acute inflammatory pain and offers an overview of the state of the art of existing delivery systems and excipients for CBD. To summarize this review, a summary of the cannabinoids and therapeutical targets of CBD will be discussed, followed by its conventional modes of administration. The transdermal route of administration and the current topical and transdermal delivery systems will also be reviewed. This review will conclude with an overview of in vivo techniques that allow the evaluation of the anti-inflammatory and analgesic potentials of these systems.

## 1. Introduction

Chronic inflammatory diseases, such as arthritis, lack a cure and represent not only an economic burden but also pose personal and social consequences for individuals living with them [1]. Moreover, people with arthritis face increased risks of experiencing mobility issues, workplace challenges, mental health issues, and overall health concerns compared to others. Despite being a persuasive health condition, arthritis remains one of the most common, affecting about 528 million people worldwide, with projections indicating an increasing rate induced by a rise in obesity and injury [2]. In addition to non-pharmacological management methods, such as physical therapy, electrical nerve stimulation, and acupuncture, arthritis pain management typically involves medications that can relieve pain and slow joint damage by limiting inflammation [2,3]. However, conventional treatments such as non-steroidal anti-inflammatory drugs (NSAIDs), opioids, disease-modifying antirheumatic drugs (DMARDs), and corticosteroids come with a lot of side effects, including gastrointestinal complications, cardiovascular diseases, hepatotoxicity, physical dependence, and immunosuppression, to only name a few [4,5,6,7,8,9,10]. The disadvantages associated with these traditional methods for pain management and the treatment of joint diseases highlight a lack at this level. The design of innovative treatments to overcome this lack is therefore required.

Phytocannabinoids originate from cannabis plants and have a historical use of over 5000 years in traditional Chinese medicine and about 3000 years in Egypt and India. In the 1970s, certain countries, including Canada and the United States, criminalized and classified this substance as illicit due to the psychotropic effects associated with tetrahydrocannabinol (THC). However, recent legalization and decriminalization of cannabis have reignited interest in it as a therapeutic agent [11]. The most abundant and pharmacologically relevant phytocannabinoids in Western countries are Δ^9^-THC and cannabidiol (CBD), mainly derived from the *Cannabis sativa* plant [12]. However, the psychotropic effects associated with THC limit its usage. Consequently, researchers primarily focus on CBD for developing new treatments as it avoids psychotropic and euphoric effects, maintains a safe profile, exhibits low potential for abuse, and offers a wide range of benefits, including antioxidative, anticonvulsive, analgesic, antitumorigenic, anxiolytic, and anti-inflammatory effects [13,14]. Therefore, CBD is a compelling candidate for treating conditions such as cancer, diabetes, neurodegenerative disorders, and inflammatory pathologies [13].

Despite CBD’s therapeutic effects, one of the main challenges regarding the use of this compound in pharmaceuticals remains its mode of administration. CBD has a low bioavailability due to its degradation by the first hepatic pass, instability in gastric conditions, high hydrophobicity, and a risk of undesirable side effects due to high concentrations in plasma [15]. Therefore, new strategies need to be developed to compensate for those characteristics. As different CBD receptors are localized in the skin, the topical and transdermal application of this drug is an interesting option, as it avoids the problems associated with conventional modes of administration and can procure high bioavailability with a steady plasma level and a good safety profile. In addition, CBD’s effects are mainly local, which contributes to decreased side effects, and the total dosage required to reach the target site is much lower [16]. Hence, this review of the literature will focus on the transdermal delivery systems and excipients of CBD for the management of acute inflammatory pain. The main endocannabinoids, phytocannabinoids, and CBD’s targets for its therapeutical effects in inflammatory diseases will be discussed. The routes with which CBD can be administered will also be addressed, as well as the existing CBD delivery systems and excipients. This review will end with some of the more commonly used in vivo models to evaluate their anti-inflammatory and analgesic properties.

## 2. The Cannabinoids

Cannabinoids constitute a substantial structural class of biological compounds capable of binding to the cannabinoid receptors (CBs). They are categorized into three groups: endocannabinoids, phytocannabinoids, and synthetic cannabinoids. This section will discuss the first two classes of cannabinoids, while synthetic cannabinoids will not be discussed further as they are irrelevant to this review. Additionally, CBD’s anti-inflammatory and analgesic effects will be addressed, as they are the most relevant for chronic pain diseases.

### 2.1. The Endocannabinoid System

The endocannabinoid system (ECS) is a crucial neuromodulatory system that plays a role in the development of the central nervous system (CNS), synaptic plasticity, and responses to both endogenous and exogenous noxious stimuli. It can also regulate bodily functions such as metabolism, food behavior, mood, anxiety, pain perception, and modulation [14]. Components of this system include at least two cannabinoid receptors (CBs), the endogenous agonists at those receptors known as endocannabinoids, and the enzymes responsible for the synthesis, transport, and degradation of those endocannabinoids [17].

CBs comprise two G protein-coupled receptors (GPCR) that mediate the effects of endogenous cannabinoid signaling: CB1 and CB2. CB1 is mainly located in the CNS, and its distribution suggests an essential role in controlling motor functions, cognition, and memory. On the other hand, CB2 is primarily located in the peripheral nerve terminals and immune cells. Its upregulation correlates with immune cell activation, suggesting a vital role in their activation and the inflammation response [18,19]. Both CBs recognize multiple agonistic and antagonistic ligands, inducing different downstream outcomes [19]. Therefore, their effects on cells mainly depend on the type of subunit α coupled to the GPCR [20]. As they typically bind to the Gα_i/0_ subunit, their activation usually inhibits adenylyl cyclase, reducing intracellular cAMP concentrations. It also inhibits voltage-dependent Ca^2+^ channels, lowering Ca^2+^ intracellular concentrations while activating mitogen-activated protein kinase (MAPK) and rectifying K^+^ channels. Therefore, their activation leads to physiological changes in the cells [17].

**Figure 1:**
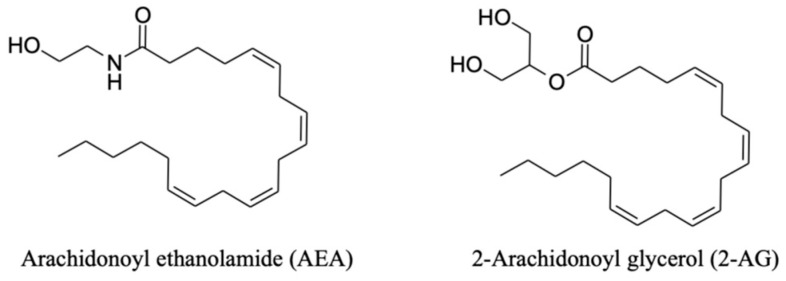
Chemical structures of the two main endocannabinoids.

Endocannabinoids are endogenous lipidic neurotransmitters that interact, among other things, with the CBs. They are synthesized in the post-synaptic terminals on demand in response to neuronal activation in a Ca^2+^-dependent manner and can subsequently bind to presynaptic CBs [18]. The two variants of arachidonic acid (AA), the 2-arachidonoyl glycerol (2-AG) and the arachidonoyl ethanolamide (AEA), also known as anandamide, are the most known and studied. However, even if their chemical structures are very similar, as shown in Figure 1, different enzymatic pathways carry out their synthesis and degradation to ensure a different physiological and pathophysiological role for these two molecules [17]. For instance, AEA has a high agonist affinity for CB1 and is almost inactive for CB2; meanwhile, 2-AG is a full agonist with moderate to low affinity for both CBs. However, both compounds interact with a large variety of receptors besides CBs, which will be further discussed in Section 2.3 [21].

### 2.2. The Phytocannabinoids

**Figure 2:**
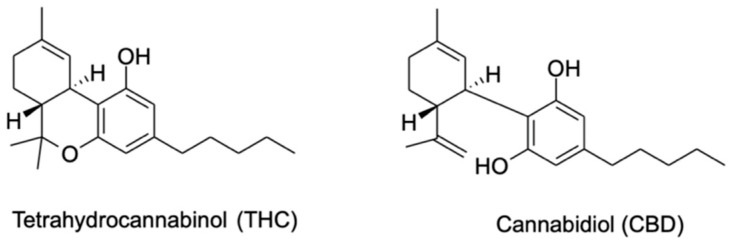
Chemical structures of the two main phytocannabinoids.

Botanical taxonomists agree that cannabis belongs to the Cannabaceae family. While more than one species exists, there appear to be three main species: *Cannabis sativa*, *Cannabis indica*, and *Cannabis ruderalis*. The first isolated and most popular in Western countries is *C. sativa* L., which contains more than 150 phytocannabinoid compounds classified into specific groups: cannabigerols (CBGs), cannabichromenes (CBCs), cannabidiols (CBDs), (−)-Δ^9^-*trans*-tetrahydrocannabinols (Δ^9^-THCs), (−)-Δ^8^-*trans*-tetrahydrocannabinols (Δ^8^-THCs), cannabicyclols (CBLs), cannabielsoins (CBEs), cannabinols (CBNs), Δ^9^-tetrahydrocannabivarins (THCVs), and cannabitriols (CBTs) [12,22,23]. Among these, the most abundant phytocannabinoids are Δ^9^-THC and CBD, sharing a similar chemical structure, as shown in Figure 2 [13,24]. The biosynthesis of these compounds begins with their acidic forms, tetrahydrocannabinolic acid (Δ^9^-THCA) and cannabidiolic acid (CBDA), which spontaneously convert into their final form due to their poor oxidative stability [12].

### 2.3. The Anti-Inflammatory and Analgesic Effects of CBD

**Figure 3:**
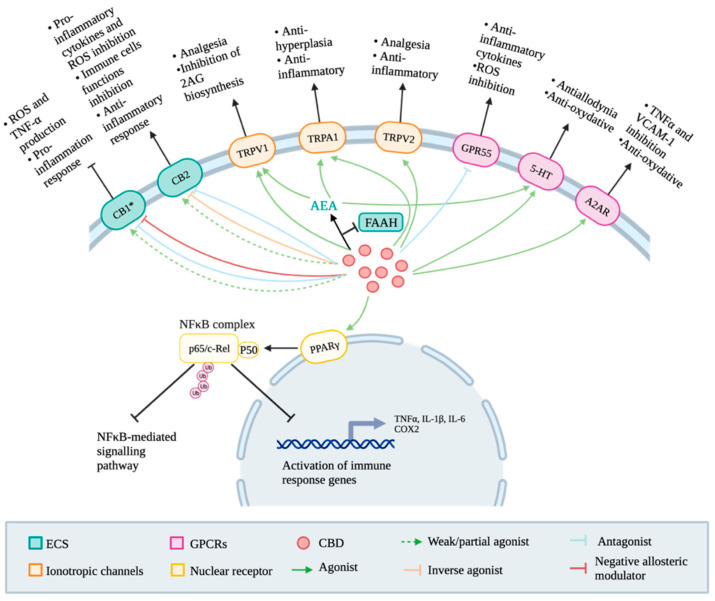
Simplified overview of the main anti-inflammatory and analgesic effects of cannabidiol (CBD).

Although the discovery of CBD predates that of THC, this compound has been less extensively studied due to its lack of psychotropic effects. However, the recent regain of interest in CBD as a pharmacological compound has prompted professionals and researchers to look deeper into the molecular pathways underlying its various therapeutic effects. Consequently, the data regarding its mechanisms of action as a therapeutic agent are now overflowing and sometimes incongruous [20]. Therefore, this section provides a comprehensive review of the main pathways underlying the therapeutical potential of CBD as an anti-inflammatory and analgesic agent. Additionally, a schematic representation (Figure 3) summarizing CBD’s targets is included at the beginning of this section to enhance comprehension.

#### 2.3.1. The Endocannabinoid System

Initial reports demonstrated that CBD is a poor competitive of endogenous ligands at the orthosteric site of CBs, suggesting that CBD mainly acts in a CBs-independent manner. Further studies suggest that CBD can act directly and indirectly on the ECS [20]. 

The activation of CB1 predominantly induces reactive oxygen species (ROS) production and downstream synthesis of tumour necrosis factor-α (TNF-α), which contribute to a pro-inflammatory response [25]. Therefore, CBD exhibits a very low agonistic affinity for this receptor, which could also explain the lack of psychotropic activity associated with this compound [20]. Hence, the antagonistic activity and negative allosteric modulation of CB1 by CBD might be partially responsible for inhibiting the inflammatory response. However, this effect is weak and is criticized by research groups. Some groups support that CBD does not bind to CB1 and that the anti-inflammatory response CBs-dependant is most likely associated with CB2 activation [20,25], even if the signaling pathways associated with this receptor activation are far less characterized than those of CB1 [26]. 

Mice lacking the CB2 receptor have an amplified inflammatory phenotype, indicating this receptor’s importance in anti-inflammatory mechanisms. The stimulation of CB2 inhibits the release of pro-inflammatory cytokines, ROS, and the proliferation, migration, and differentiation of various immune cells [25,27]. However, the pharmacokinetic studies suggested that CBD may be a very low agonist of CB2 because it seems to have a detectable effect only in the micromolar range. However, despite its low agonistic affinity for CB2, CBD appears to have a biological effect at reasonable concentrations in the nanomolar range [28]. This was later proven due to its inverse agonist and antagonist activities [25,28]. Evidence shows that the inverse agonistic mechanism could inhibit immune cell migration and decrease inflammation symptoms [28,29]. Lunn’s team has assessed that the binding of a selective inverse agonist to CB2 could inhibit the migration of leukocytes associated with the binding of 2-AG, a very good agonist of this receptor, and because the injection of this inverse agonist could reduce leukocyte trafficking in rodents [29]. CBD’s indirect modulation of CB2 could also explain its anti-inflammatory effect [25]. CBD has been shown to inhibit the cellular uptake of AEA and the activity of the fatty acid amide hydrolase (FAAH), which is responsible for the hydrolysis of AEA. Therefore, an increase in the concentrations of this anti-inflammatory effector can be seen in cells in the presence of CBD [18]. 

Endocannabinoids can promote both pro-inflammatory and anti-inflammatory responses. On the one hand, AEA was found to downregulate ROS production, the release of pro-inflammatory mediators and immune cell functions, such as leucocyte migration, and increase the release of anti-inflammatory cytokine IL-10. Conversely, 2-AG seems to enhance B and T cells, dendritic cells, eosinophils, monocytes, and natural killer cells recruitment and functions, production of ROS, pro-inflammatory cytokines, and autacoids [26,30]. Even though 2-AG could be accountable for the pro-inflammatory effects, the endocannabinoids’ metabolites could also play a significant role in immune cells’ positive modulation [30]. The metabolization of endocannabinoids by eicosanoid biosynthetic enzymes increases AA levels, promoting the biosynthesis of eicosanoids, such as PGH2-EA, PGE2-GE, and leukotriene. Hence, those bioactive lipids play a significant role in the development of the inflammatory response [30], and inhibition of their biosynthesis could help improve inflammation.

#### 2.3.2. GPCRs

CBD can also exert its anti-inflammatory and analgesic properties independently of the ECS by modulating various GPCRs and signaling pathways. Potential targets for CBD include the serotonin 1a receptor (5HT1a), the adenosine A2A receptor (A2AR), and the G protein-coupled receptor 55 (GPR55). In the serotoninergic system, CBD has demonstrated the ability to attenuate mechanical allodynia by regulating neurotransmitter release and neuronal excitability [18]. Additionally, when activated by CBD or AEA, 5HT1a can have antioxidant effects by capturing ROS, potentially contributing to the indirect improvement of inflammation [25].

In counterpart, the activated A2AR exhibits anti-inflammatory proprieties, reducing levels of TNFα and vascular cell adhesion protein 1 (VCAM-1), which are implicated in immune cell migration [25]. CBD can also inhibit the cellular uptake of adenosine, increasing its endogenous content for A2AR activation [18]. A2AR may also possess antioxidative properties, helping to reduce oxidative stress [25]. 

The GPR55 receptor, predominantly found in nervous and immune cells, may also play a role in immune functions. CBD has been shown to exert an antagonistic activity on GPR55, but the exact mechanism underlying this pathway remained unclear. CBD may modulate this receptor’s activity via the modulation of endocannabinoid levels [25]. However, observations from Staton’s team suggest that GPR55 knockout mice exhibit high levels of anti-inflammatory cytokines and reduce hyperalgesia, while high receptor expression inhibits ROS production [31]. Therefore, the final effect of this receptor’s activation may depend on the dominating effect of CBD’s direct or indirect activation [25]. 

#### 2.3.3. Ion Channels

TRP channels, such as TRP vanilloid 1/2 (TRPV1/2) and TRP ankyrin 1 (TRPA1), are members of a large family of ionotropic channels involved in transduction in response to diverse physical and chemical stimuli by modulating calcium levels [32]. CBD can activate them directly or indirectly, considering that one of the endogenous ligands of TRPV1 is AEA. CBD’s action in reducing oxidative stress can also ensure the presence of the active form of this receptor [25]. However, activating these receptors is generally associated with inflammation and pain, but some agonists, like CBD and AEA, can induce paradoxical analgesia by desensitizing these receptors [32]. Their activation may also inhibit the biosynthesis of 2-AG, reducing the levels of pro-inflammatory endocannabinoids [25]. Therefore, CBD’s sustained activation of TRPV1/2 and TRPA1, along with increased AEA, contributes to analgesic, anti-inflammatory, and anti-hyperalgesic effects, aiding in managing inflammatory pain [25,32,33]. 

#### 2.3.4. Nuclear Receptor

The peroxisome proliferator-activated receptor γ (PPARγ) is a member of the nuclear receptor superfamily of ligand-inducible transcription factors and plays a vital role in the negative regulation of inflammation. Upon activation by agonists such as CBD and endocannabinoids, it can modulate the ubiquitination of cytoplasmic and nuclear NFκB p65 subunit by interacting with a ubiquitin-conjugating enzyme, UbcH3 [25,34]. The addition of polyubiquitin to this subunit induces subsequent proteasomal degradation, inhibiting the NFκB-mediated inflammatory signaling pathway, downstream gene expression of cyclooxygenase 2 (COX2), and the expression of pro-inflammatory cytokines such as TNF-α, IL-1β, and IL-6 (25). 

As seen in this last section, CBD has the potential to regulate a wide variety of receptors, ionotropic channels, and enzymes, as well as the levels of endocannabinoids. The main direct and indirect targets and CBD’s mechanisms of action are summarized in Figure 3.

## 3. Administration of CBD

While CBD offers therapeutic benefits, its effective use in pharmaceuticals faces significant hurdles, primarily concerning its administration. CBD’s low bioavailability stems from its degradation during the initial hepatic pass, instability in gastric environments, high hydrophobicity, and the potential for undesirable side effects due to elevated plasma concentrations [15]. Hence, novel approaches must be developed to address these inherent traits. This section will discuss a review of the products containing cannabinoids authorized by the regulation agencies of Western countries, the modes of administration with which CBD can be consumed in a user-friendly setting and will conclude with transdermal CBD systems in the development phase.

### 3.1. Cannabinoids-Based Products Approved in Western World

#### 3.1.1. Health Canada in Canada

As of today, the regulatory agency of Canada, namely Health Canada, has approved three cannabinoids-based drugs, with only two still being on the market. Sativex^®^ (GW Pharma Ltd., Cambridge, UK), also known as Nabiximols, comes as an oromucosal spray and contains a mix of THC (Tetranabinex^®^) and CBD (Nabidiolex^®^) in a 1:1 ratio. It has been used as a treatment for symptomatic relief of spasticity in adult patients with multiple sclerosis (MS) since 2005 in Canada [14,35,36]. Marinol^®^ (Solvay Pharmaceuticals, Inc., Brussels, Belgium), in which the therapeutic agent is a synthetic THC (dronabinol), was used to treat loss of appetite in acquired immune deficiency syndrome patients (AIDS) and nausea from chemotherapy in the 1990s. However, the manufacturer voluntarily withdrew those oral capsules from the Canadian market in 2012 and they are no longer available in Canada. The last approved treatment is Nabilone (Cesamet™; Bausch Health Compagnies Inc., Laval, QC, Canada), which contains a synthetic analogue of THC. This treatment has been available as oral capsules since 1982 and is used to reduce nausea and vomiting associated with cancer chemotherapy in patients who have failed to respond adequately to conventional antiemetic treatment [35].

#### 3.1.2. Food and Drugs Administration (FDA) in the United States

The development of therapeutic products containing cannabinoids is progressing rapidly, and currently, four are authorized by the FDA for use in the market. Epidiolex^®^ (GW Pharma Ltd., Cambridge, UK), approved in 2018, is a CBD-based drug administrated as an oral solution for the treatment of two rare and severe forms of childhood epilepsy, namely Lennox-Gastaut syndrome and Dravet syndrome in patients one-year-old and older [13,37]. Meanwhile, two drugs containing dronabinol as their active ingredient, Marinol^®^ and Syndros^®^ (Benuvia Therapeutics, Round Rock, TX, USA), obtained approval in 1985 and 2016, respectively, for the same purpose as in Canada [13,38,39,40]. Nabilone (Cesamet™; Bausch Health Compagnies Inc., Laval, QC, Canada) also gained approval in 1985 in the US for chemotherapy patients [13,40].

#### 3.1.3. European Medicines Agency (EMA) in European Countries

There are currently two products containing cannabinoids that are approved by the EMA. Epidyolex^®^ (Jazz Pharmaceuticals Ireland Ltd., Dublin, Ireland) has been used as intended by the US since 2014 for the Davet syndrome and since 2019 for the Lennox–Gastaut syndrome. It is also used for treating tuberculosis complex as an adjunct therapy to other treatments in patients two years old and above [41]. Other authorized products include Sativex^®^, which holds marketing authorization in more than 18 EU member states for the treatment of MS [42].

Despite these great products, work remains to be done to fully exploit the potential of CBD while minimizing the side effects associated with higher doses. To achieve this objective, it is essential to understand the different administration routes and their advantages and limitations.

### 3.2. Delivery Routes and Bioavailability of CBD

The bioavailability of CBD, which impacts the efficacity of the treatment, varies significantly based on the delivery route. The parameters influencing a drug’s capacity to bind to its receptors are its physiology, dissolution, stability, permeation, and metabolism. As mentioned, CBD is highly lipophilic, with a log *p*-value ranging from 6–7, and hydrophobic, exhibiting a miscibility of 2–5 μg/mL in water. It is also light- and temperature-sensitive with poor oxidative stability [16]. Therefore, it is essential to consider those characteristics when engineering new delivery methods. 

#### 3.2.1. Oral Administration

The oral route is one of the most explored modes of administration for CBD but has encountered some drawbacks due to its low bioavailability. The issue is partly attributed to the highly acidic stomach environment and the hepatic first-pass metabolism, which inactivates about 95% of CBD. Other factors include slow and erratic absorption, weak therapeutic efficacity, and challenges associated with administrative dosage. Consequently, only 6–20% of the dose has been reported in blood plasma after oral administration and was only detectable for a short period with a peak at 1–4 h post-administration and a significant decrease after 6 h [16]. Therefore, patients need to take repeated doses quickly, which could limit its acceptance and patient compliance. Furthermore, the repeated dosage could also be problematic for chemotherapy patients due to nausea and vomiting [15]. 

#### 3.2.2. Sublingual Administration

The sublingual placement could avoid degradation by hepatic enzymes and gut acidity. In addition, it procures fast absorption into the blood and is easy to use. However, this route is associated with a considerable increase in saliva production, prompting the swallowing of the product. Thus, the benefits associated with sublingual applications are lost [15]. 

#### 3.2.3. Delivery by Inhalation

CBD inhalation, through smoking or vaporization, has a bioavailability of 2–56% and up to 50%, respectively. However, inhalation is associated with adverse pulmonary impacts and inconsistent effects depending on the length, volume, duration, and inhalation rate [16].

#### 3.2.4. Alternatives Routes: Intranasal, Rectal, and Intravenous Routes

Alternative routes of CBD administration, such as intranasal, rectal, and intravenous, allow rapid absorption into the systemic circulation due to the presence of several vasculature structures and the nature of the tissues involved. This approach avoids hepatic metabolism and gastric instability, enhancing bioavailability [16]. However, significant challenges are associated with these routes. Delivering therapeutical doses of CBD via the nasal epithelium is difficult due to the compound’s lipophilicity. It can also cause nasal mucosa irritation and mucociliary clearance. Therefore, potent drugs are more suitable for this method of administration in comparison to chronically administered doses [15]. Despite the drug being directly injected into the bloodstream in the intravenous route, this method is however undesirable due to its invasive nature, the increased risk of infection, and the lack of compliance in patients [16].

#### 3.2.5. Topical and Transdermal Administration

**Figure 4:**
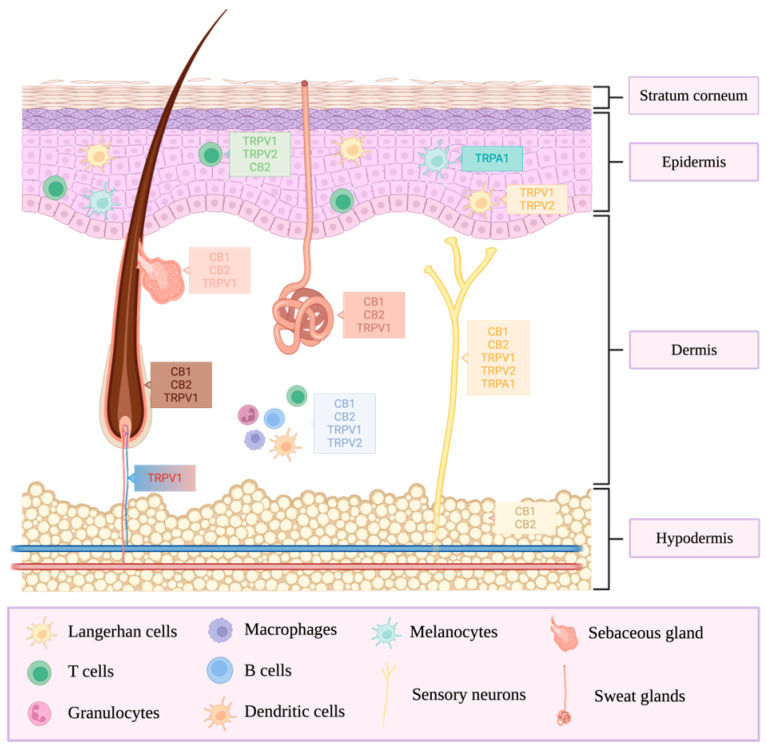
Cannabidiol receptors in the skin. CB1/2: cannabinoid receptor 1/2, TRPV1/2: transient receptor potential vanilloid 1/2, TRPA1: transient receptor potential ankyrin 1.

Different CBD receptors are localized in the skin, as represented in Figure 4, indicating that it could directly act in this tissue. Therefore, a topical and transdermal application could be a viable alternative for CBD delivery. These application methods are of high interest because they avoid the problems associated with conventional modes of administration and can procure high bioavailability with a steady plasma level and a good safety profile. In addition, CBD’s effects are mainly local, which contributes to decreased side effects, and the total dosage required to reach the target site is much lower [16]. However, transdermal drug delivery still encounters challenges, such as skin permeation. Therefore, to develop effective cutaneous drug delivery technologies for CBD, it is essential first to understand the skin’s structure.

The skin is the barrier between the body and its environment. It comprises three layers: the hypodermis, the dermis, and the epidermis. The stratum corneum (SC) is the outer layer of the epidermis. It comprises keratin-filled corneocytes enclosed in a lipidic matrix mostly composed of ceramides, free fatty acids, and cholesterols [15,43]. It essentially protects the other skin layers and contributes highly to skin permeability. Therefore, only a few components can pass through this layer, such as heat, a few fluids, and low-weight molecules, by paracellular (intercellular), transcellular (intracellular), or trans-appendageal pathways (e.g., hair follicles and sweat glands). Therefore, the penetration of therapeutical agents through this layer is one of the limiting steps for topical and transdermal applications. Another challenge associated with this route is the hydrophilic layers of skin situated below the SC. Hence, drugs must be lipophilic enough to pass through the SC and hydrophilic enough to penetrate the other skin layers [15].

Drugs must respect specific characteristics to ensure adequate permeation and diffusion into the skin. Ideally, it must be moderately lipophilic, have a molecular weight lower than 500 g/mol, a melting point lower than 250 °C, a high potency, and a p-log value between 1–3 [15,43]. Therefore, CBD’s high lipophilicity makes it an inadequate candidate for this application because it tends to accumulate in the SC instead of diffusing through other skin layers. Consequently, strategies are being developed to enhance the transdermal delivery of such compounds to overcome these unfavorable characteristics. Among them are the addition of chemical penetration enhancers, ions pairing, eutectic systems, physical permeation enhancers, and the encapsulation in lipidic or polymeric delivery systems (nanoparticles (NPs) and nano gels, for instance), to name a few [15].

### 3.3. Technics Used to Enhance Transdermal Delivery of CBD

As mentioned previously, only two cannabinoid-based drugs have gained Health Canada approval, four have FDA approval, and two have EMA approval, with just one being truly CBD-based. This drug is available as an oral solution, resulting in limited bioavailability. Therefore, various lipidic and polymeric delivery systems, as well as different excipients, have been developed for the transdermal delivery of CBD. These systems show great promise in enhancing the effective dose delivery of lipophilic drugs like CBD, providing protective effects for therapeutic agents exhibiting poor stability, and offering targeted and controlled delivery [16]. Transdermal delivery also ensures stable blood levels over an extended period, providing prolonged pain relief for patients. Using creme, gel, ointment, or patch could also allow for easy self-administration without requiring medical assistance.

However, as research in this field advances, a comprehensive understanding of the effectiveness and benefits of transdermal CBD delivery systems is crucial. Table 1 summarizes various preclinical and clinical studies, offering valuable insights into the therapeutic properties and potential applications of transdermal CBD. As depicted in this table, CBD presents a broad spectrum of therapeutical benefits, including anti-inflammatory, analgesic, antiemetic, neuroprotection, and antioxidant properties, among others. Even if some of the conditions summarized in this table do not directly treat or manage chronic inflammation diseases, they bring valuable insight into the transdermal delivery of CBD. They could, therefore, be useful or applicable to the elaboration of CBD-based transdermal inflammatory pain disease treatments.

Ethosomes are lipid-based vesicular systems containing a high percentage of ethanol, demonstrating improvement in transdermal drug delivery with respect to other NPs types, such as liposomes. Their content in ethanol allows for improved flexibility and deformability, leading to less drug leakage during transport. It also contributes to lipid fluidization of the cells’ membrane in dermal layers for in-depth penetration of the active compound, in our case, of the CBD [61]. The excipient of the NPs is also crucial in the efficacity of a system. Carbomers, such as Carbopol^®^, Pemulen^TM^, Noveon^®^, and Polycarbophil, are the most frequently used class of gelling agent in approved topical pharmaceuticals. They stabilize suspensions and modify the rheological properties of topical formulations. Furthermore, the acrylic acid-based polymers crosslinked with polyalkenyl ethers or divinyl glycol render varying viscosity characteristics due to the resulting unique three-dimensional structure enabling hydrodynamic swelling of the gel [62]. In Lodzki’s research, the ethosomal formulation contained 3% *w*/*w* CBD and 40% *w*/*w* EtOH in a carbomer gel. This system has resulted in a significant accumulation of the drug in the skin and the underlying muscle with a steady-state plasma level after 24 h, which lasts at least until the end of the experiment. They also assess the anti-inflammatory potential in a carrageenan-induced aseptic paw inflammation in male IRC mice, where inflammation was successfully prevented in the treated paws. Moreover, the paw thickness of CBD-pretreated mice was statistically different from non-pretreated mice’s starting 1 h post-injection [1].

In Hammell’s study, CBD at 1 and 10% was added to a Carbopol^®^ 980 polymer (0.9% *w*/*w*) to evaluate inflammation and pain-related behaviors in a complete Freud’s adjuvant (CFA)-induced arthritis model in male Sprague Dawley rats. The results indicated that the CBD gel is an effective treatment for reducing inflammation and hypersensitivity associated with the arthritic model. The 6.2 mg/day dose optimally reduced swelling in the knee joint, while the 62.3 mg/day did not yield additional improvement. Following the CFA injection, synovial membrane thickening was also observed, where the transdermal application of both doses significantly reduced this pathological change. They also observed an improvement in pain score as an indirect measure of joint inflammation. Both doses led to optimal paw withdrawn latency in response to noxious heat stimuli [44].

Transcutol, or diethylene glycol monoethyl ether, is an excipient and surfactant widely used in cosmetics and pharmaceutics to increase the drug penetration of formulations. Furthermore, this compound has gained a lot of attraction due to its optimal properties: nontoxicity, biocompatibility with the skin, miscibility with polar and non-polar solvent, and optimal solubilizing properties for many drugs [63]. Transcutol has been investigated as an excipient for the transdermal delivery of CBD by Gonzalez-Cuevas’s team for the prevention of relapse to drug use. Hence, CBD levels in the plasma and brain of male Winstar rats were evaluated. A significant dose-dependent CBD level was detectable in the plasma and brain on treatment day 7, as well as 3 days post-treatment, which could be explained by a skin reservoir effect of the formulation [45].

Liput et al. have investigated the role of transdermal CBD in binge alcohol-induced neurodegeneration in a model of male Sprague Dawley rats of alcohol use disorder. The 5% gel formulation has neuroprotective effects, while the 1.0% and 2.5% were ineffective. The mean CBD plasma concentration on day 3 for the 5% group was then evaluated at 100 ng/mL, which suggests that this delivery system could efficiently deliver therapeutical doses of CBD across the skin barrier [46].

Cellulose and cellulose derivates are biocompatible, non-toxic, and biodegradable polymers that can react with different molecules through esterification, etherification, and oxidation, affording derivates with excellent properties. Cyclodextrin (CD), a class of cyclic oligosaccharides, encompasses important features, including excellent biocompatibility and chemical stability, high hydrophilicity, and biodegradability with unique amphiphilic and transport properties [64]. Due to their structure, CDs can also form inclusion (host-guest) supramolecular complexes with a large variety of molecular guests. Furthermore, the spatial arrangement of CDs leads to a relatively hydrophobic cavity with a hydrophilic external surface, enabling the thread of a broad range of hydrophobic compounds such as CBD. The resulting complex is highly stable, which contributes to the improvement of the guest properties like solubility enhancement and antioxidant control. Therefore, excipients derived from cellulose and CDs have been extensively used in pharmaceuticals due to their specific binding properties suitable for controlled and sustained drug delivery [64]. Hence, a novel drug delivery platform for the topical delivery of CBD based on CBD/β-cyclodextrin complexes within biocompatible cryogel was documented by Momekova et al. for the treatment of primary skin tumors or cutaneous metastases. The team synthesized a cryogel through the photochemical crosslinking of 2-hydroxyethyl cellulose and β-cyclodextrin in a frozen aqueous system, which was subsequently loaded with CBD. In this study, the CBD spontaneously forms an inclusion complex with β-CD at a 1:1 ratio, where an increase in β-CD concentration leads to an increased fraction of tightly bound CBD. This could explain the observed sustained CBD release from β-CD-containing cryogel matrices. Moreover, their team observed a bi-phasic release behavior with an initial burst in the first 3 h, which could be explained by the CBD absorbed in the cryogel walls, followed by a slower CBD release from the guest-host complex [47].

Chitosan-based hydrogels are promising naturally derived polysaccharides for pharmaceutical applications. This multifunctional hydrogel can encapsulate, carry, and release the drug with good biocompatibility, biodegradability, and absence of immunogenicity [65]. Moreover, chitosan hydrogels are easy to handle due to their mucoadhesive properties, providing prolonged contact with the site of action. However, despite their advantages, chitosan hydrogels cannot encapsulate lipophilic substances and could lead to potential toxicity because of the initial burst release. As for the nanoemulsions, they usually present with low viscosity and low spreadability, making them less suitable for transdermal application. The incorporation of nanoemulsions (NEs) within the chitosan hydrogel, therefore, has gained interest as the resulting system possesses different characteristics from the initial colloid systems. They can encapsulate both lipophilic and hydrophilic drugs, have enhanced stability and texture, provide controlled release, improve biocompatibility, and release profile from the incorporated drug [51]. Demisli et al. have developed a system of encapsulated CBD in oil-in-water (O/W) NEs and NEs-filled hydrogel for the treatment of various conditions, including epilepsy, psychiatric and skin conditions, pain, and inflammation. The NEs were composed of water, Transcutol, and a mixture of surfactants (Labrasol, Tween 80, and Maisine) with an immiscible phase of isopropyl tetradecanoate and organic extra virgin olive oil. The resulting O/W NEs are thermodynamically metastable but kinetically stable in the nano-range, composed of oil droplets dispersed in an aqueous medium and stabilized by surfactant molecules. Ex vivo and in vitro studies have also shown that the developed system effectively delivers CBD across the skin while maintaining good biocompatibility [51].

Alginate is a natural and versatile polymer of marine origin with good biocompatibility, ease of handling, nontoxicity, mild gelation properties, and low cost. Alginate is hydrophilic and water-soluble, thickening in neutral conditions, and can form a hydrogel in the presence of polyvalent cations [66]. Therefore, Zheng’s team developed a CBD-containing alginate-based hydrogel enriched in Zn^2+^ (CBD/alg@Zn) to promote wound healing. The hydrogel was synthesized by ion crosslinking of the Zn^2+^ and the alginate polymer, and the CBD was embedded in the hydrogel simultaneously. The team observed that the CBD/alg@Zn system has antioxidant activity and could reduce the inflammatory response while exhibiting good biocompatibility [52].

Sepigel 305 comprises a polyacrylamide gelling agent, a non-ionic emulsifier (polyoxyethylene 7 lauryl ether), and a fatty oil (isoparaffin). This compound led to medium to high viscosity formulation and allows the incorporation of both hydrophilic and lipophilic substances with good stability [67]. Vanti et al. developed a CBD-loaded O/A ME formulated as a microemulgel for treating skin diseases, including eczema, psoriasis, pruritus, and inflammatory conditions. The system consists of Solutol HS 15 (20%, surfactant), Transcutol P (9%, cosolvent), isopropyl myristate (5%, oil phase), water (66%), and 1% *w*/*w* CBD, which has been thickened with Sepigel 305. The resulting system shows excellent homogeneity of the ME droplets, high stability in pH viscosity, and CBD content. The release and permeation studies they performed on a skin-PAMPA model and through a rabbit ear skin also showed controlled release and absorption of the CBD, resulting in good retention in the skin layers [53].

Eudragit RL100 is a co-polymer containing methyl methacrylate and ethyl acrylate with quaternary ammonium groups and less methylacrylate acid. This polymer is associated with sustainable drug release with high permeability [67]. Casiraghi et al. tested the impact of different delivery systems on the skin permeation of CBD. The two developed systems that show greater promise were a propylene glycol/water mixture (80/20 *v*/*v*), a 3D printed Eudragit RL (59.4% *w*/*w*), and tributyl citrate (39.6% *w*/*w*) CBD patch. The transformation of the solution into hydrophilic gel did not seem to affect the performance in terms of permeation and led to a reduction of the amount of CBD retained in the SC. They found that the transdermal patch has lower dosing and unfavorable thermodynamic conditions. However, this transdermal system provided a comparable skin retention of the CBD [54].

Stinchcomb’s team studied the skin permeation of different cannabinoids, including ∆^8^-THC, CBD, and CBN, as they are good candidates for combination therapy for chemotherapy side effects management. The excipients they used are mineral oil with 7:3 propylene glycol:water or 4:5:4 propylene glycol:water:ethanol. They found that 30–33% ethanol concentration significantly enhanced the flux of ∆^8^-THC and CBD [55].

Radwan-Pragłowska et al. developed a novel transdermal delivery system of CBD by functionalizing chitosan with ZnO NPs for treatment-resistant epilepsy. This system has superior porosity, excellent swelling properties, mechanical durability, drug-loading capacity, and a prolonged release of CBD. Moreover, this system had conductive properties, which makes its application possible by iontophoresis. The CBD-loaded chitosan-ZnO system also shows good biocompatibility [56].

Silica particles have gained popularity in the biomedical field because of their stability, hydrophobicity, biocompatibility, ease of functionalization, large specific surface area, high pore volume with adjustable pore size, controlled particle size, high loading capacity for drugs, controllable drug release and ease of synthesis and processing. The chemical and physical functionalization of the particles also allows the encapsulation of both hydrophilic and hydrophobic drugs [68]. Khabir et al. encapsulated CBD within a nanoporous organosilica matrix. The resulting system can stabilize CBD for a period of up to 18 weeks with only a small product degradation. However, the transdermal penetration studies demonstrate the presence of CBD within the dermis layer of the skin below therapeutically relevant concentrations. They also embedded the CBD–Silica particles within a thin PVA film. The results suggest that this system can release stable CBD with an enhanced dissolution profile compared to the particles alone and pure CBD. A higher concentration of CBD is also measured below the SC when the formulation is incorporated in PVA films than with pure CBD, consistent with the enhanced dissolution profile. However, further optimization of the dose and transdermal penetration are required for this formulation to be successfully implemented [57].

Park et al. tested various microemulsions (ME), including Capryol 90 as oil phase, Tween 80, Solutol HS15, Procetyl AWS, and Cremophor RH40 as surfactants, ethanol as cosurfactant, and distilled water as the aqueous phase for the transdermal delivery of acidic cannabinoids. One of the MEs, composed of 1.0% (*w*/*w*) of cannabinoids, 5% (*w*/*w*) of Capryol 90, 44% (*w*/*w*) S_mix_ (2:1, Procetyl AWS and ethanol), and 50.0% (*w*/*w*) of distilled water, showed a significant improvement in transmembrane flux, permeability coefficient, and enhancement ratio [58].

In addition to the main polymeric or excipient used in the formulations above, other compounds can be employed to facilitate the penetration of CBD into the skin. Chemical permeation enhancers (CPEs), such as propylene glycol, ethanol, and labrasol, are often combined with these systems to enhance skin permeation by interacting with the skin barrier components to temporarily reduce its permeability or modify the formulation properties to increase the active component uptake into the skin [43]. These enhancers should possess a high specificity, non-toxic, non-irritating, and non-allergenic effects, a fast, predictable, and reproducible response, unidirectionality effects, compatibility with the drug and formulation compounds, and acceptance for use in the cosmetic domain [43]. CPEs that affect the skin barrier can act in three different ways. They can alter the lipids by either incorporating lipid lamellae to fluidize the lipid chains or by adding enhancer-rich, more permeable domains in the lipid lamellae. The second mechanism is the alteration of the proteinic components of the SC either by changing the keratin conformation, causing swelling and hydration, or by altering the cohesion between corneocytes. The last method is altering the solvent nature of the tissue, which impacts the partitioning of the drug from the formulation into the SC [43]. It is also important to note that most enhancers can act via multiple mechanisms. However, one of the main challenges associated with using CPE is finding potent but safe enhancers, as when they disrupt the skin barrier, they often also affect cell viability and cause toxicity. Therefore, different strategies can increase the potency while minimizing unwanted side effects, such as skin irritation and toxicity. The first strategy consists of utilizing a synergistic combination of enhancers that have, for example, different mechanisms of action. Consequently, the concentration of enhancers can be lower than usual, decreasing toxicity while maintaining efficacity. The other strategy consists of biodegradable enhancers. Such enhancers will interact with the SC to increase the permeation of the drug and be transformed by enzymes of the skin into a non-toxic compound when they reach deeper viable strata (e.g., amino acid-based amphiphiles linked by an ester bound to a fatty alcohol are degraded by the esterases of the skin) [43].

## 4. In Vivo Models for Assessing Anti-inflammatory and Analgesic Properties

While assessing a new drug’s biological effects and efficacity, it is crucial to carefully choose a representative in vivo model so that the conclusions drawn in those studies are as accurate as possible and can be extrapolated to humans. Finding a safe and effective drug to control inflammation has been a challenge, and therefore, many animal models have been developed to evaluate the drug’s anti-inflammatory properties. Hence, this section will focus on paw edema models of inflammation in mice/rats since they are the most relevant to this review. Table 2 at the end of this section will summarize the advantages and limitations of each technique.

Murine models of paw inflammation are subdivided into two categories: acute inflammation and chronic inflammation. Both models are used to evaluate drugs that modulate erythema, blood vessel permeability, leukocyte infiltration, chemotaxis, and phagocytosis and assess some of the proprieties of the drugs, such as antipyretic, analgesic, and anti-inflammatory actions. However, the duration and intensity of inflammation vary between the two. Acute inflammation is more of a transient and restrained reaction, while chronic inflammation models persist for extended periods and usually harmonize the disease process [69].

### 4.1. Carrageenan-Induced Edema

Carrageenan-induced paw edema is a widely used and well-established model of acute inflammation to screen the anti-inflammatory properties of various natural and synthetic drugs. In addition, it demonstrates good reproducibility and acts on several inflammatory pathways, making it a good model for preliminary analyses. Furthermore, this non-antigenic phlogistic agent acts locally in a bi-phasic manner, enabling the model to predict a probable biological target of the tested drug [69]. In the first hour following carrageenan injection, the injection trauma and the release of inflammatory mediators, such as histamine, serotonin, and bradykinin, account for most of the early acute phase of inflammation. The second phase, which occurs around 3 h post-injection, is mainly mediated by PGs and neutrophil infiltration, which release free radicals and pro-inflammatory cytokines, such as TNF-α et IL-1β [70]. This phase also corresponds to the maximal swelling and subsides by 24 h [71]. However, the concentration of carrageenan injected highly affects the edema response, whereas the maximum response is too arduous to inhibit. Hence, the solution must be precisely prepared to avoid deviation from the expected response [69].

### 4.2. Histamine/Serotonin-Induced Edema

As seen in the carrageenan-induced edema, histamine and 5HT are two essential modulators of the early phase of inflammation. They enhance vasodilatation, vascular permeability, edema formation, leukocyte infiltration, and modulate other inflammatory mediator levels, causing acute inflammation [72]. Their injection causes an influx of lymph and plasma proteins into the extracellular matrix, which is responsible for the local edema. The binding of histamine to the receptor H1 also causes the contraction and separation of endothelial cells, which increases vascular permeability. Histamine also causes the release of neuropeptides and PGs, causing the phenotype of hyperalgesia and inflammation associated with histamine [69]. However, those models are mainly used to evaluate the acute anti-inflammatory effects of drugs that act by inhibiting histamine or serotonin. It can also be used, complementary to the carrageenan model, to confirm the biological target and efficacity of the first phase of inflammation. However, the effects of histamine and 5HT are minimal and temporary [73].

### 4.3. Bradykinin-Induced Edema

Bradykinin is also a crucial mediator of the early acute inflammation response. Along with histamine and 5HT, it acts as a vasodilator in swelling and inflammation. In this model, the injection of bradykinin stimulates the phospholipase A2 activity, promoting PG biosynthesis [69]. It can also activate TRPA1, triggering the sensation of pain. In addition, the activation of bradykinin receptors also stimulates the production of other inflammatory mediators and cytokines [74]. Therefore, it can be used as a secondary model for carrageenan-induced edema to evaluate drugs inhibiting PGs. However, it only produces mild short-term edema and cannot be used to assess histamine/5HT inhibitors [69].

### 4.4. Dextran-Induced Edema

The dextran-induced paw edema model is used to study the acute anti-inflammatory properties of drugs. It increases vascular permeability, the activation of kinins, and the release of histamine and 5HT from mast cells, leading to osmotic edema [75]. The dextran effect is biphasic; the first phase, which happens in the first hour, is characterized by extravasation and edema formation due to histamine liberation, increased vascular permeability, and increased blood flow to the inflammation site. The second phase, which takes place between 1–6 h, is accompanied by the enhancement of free radicals, bradykinins, PGE2, and cytokines levels. In addition, COX2 levels, the enzyme responsible for the biosynthesizing of PGs and leukotrienes, also seem to be increased in paws injected with dextran [76]. In sum, this model is suitable for evaluating drugs that act on histamine, 5HT, and bradykinins and can be used to reinforce the results of carrageenan-induced inflammation [69].

### 4.5. Liposaccharide-Induced Edema

In response to the binding of LPS to its receptor, the toll-like receptor 4, a cytokines liberation (TNF-α, IL1-β, and IL-6) with NFκB nuclear translocation and myeloperoxidase activity, is observed along the edema response [77]. Furthermore, this acute inflammation response is localized, and the peak of inflammation is observable at 1–3 h. Therefore, this model adequately assesses drugs’ efficacity in modulating cytokine levels. It is also suitable to assess anti-hyperalgesia drugs’ properties [69].

### 4.6. Formalin-Induced Edema

Injection of small doses of formalin in rodent paws produces a biphasic behavioral response and is frequently used as a model of persistent pain. In the initial pain burst arising about 5–10 min post-injection, licking, biting, and elevation of the injected paw is observed, followed by a 5–10 min recess moment where relatively normal behavior is observed. Then, the second pain burst occurs around 30 min later. The initial pain response is thought to be associated with the activation of nociceptive neurons, while the second pain response is likely associated with inflammation within the tissue [78].

### 4.7. Complete Freud’s Adjuvant (CFA)-Induced Edema

CFA-induced edema is a well-studied model of chronic inflammation and arthritic modifications involving various systemic changes. This model is characterized by leukocyte infiltration, increased levels of chemokines, cytokines (TNF-α, IL1-β), ROS, cartilage and bone destruction, swelling, and deformations. Therefore, it involves immune–inflammatory components, where the primary lesions (injected) and the secondary lesions (non-injected) represent human inflammation and arthritis, respectively. It is then possible to assess acute, chronic, and immune inflammation and arthritic conditions with this model [69]. Following the injection of CFA into mice’s footpad, edema gradually appears in the early stage of inflammation to reach maximal swelling at 24 h post-injection and becomes more constant within two weeks [69]. Therefore, this model allows measurement of alterations such as paw volume with a plethysmometer and pain threshold with a Von Frey fiber and Modified Hargreaves apparatus. However, because the experiment is longer and more painful than the other models, it involves more animal stress [69].

## 5. Conclusions and Future Perspectives

In conclusion, this literature review has provided a comprehensive overview of the role of transdermal CBD in managing chronic inflammatory pain, as observed in arthritis, along with cannabinoid-based treatments approved by the Canadian, American, and European pharmaceuticals regulation authorities. Transdermal delivery of CBD shows great promise to address a broad range of pathologies, including acute inflammatory pain diseases. Various CBD nanoformulations and CBD-loaded excipients show great potential in addressing critical issues such as bioavailability, stability, and controlled release, offering a potential novel treatment for chronic inflammatory pain. This review has drawn attention to the most used in vivo testing methods that can be utilized to determine the anti-inflammatory and analgesic performances of CBD-loaded formulations. As a lot of work remains to be done, researchers and scientists must remain creative, collaborative, and adaptive to ensure that the CBD formulations for transdermal delivery can be translated into tangible clinical benefits.

## Figures and Tables

**Table 1:** Current studies on transdermal application of CBD.

Delivery Systems and/or Excipient	Forms	CBD Concentrations/Quantities	Applications	References
Ethosome in a carbomer gel	Gel	3% *w*/*w*	Chronic inflammatory disease treatment (rheumatoid arthritis)	[1]
Carbopol^®^ 980		1 and 10%	Arthritis treatment	[44]
Transcutol		2.5 g CBD/100 g gel	Drug addiction treatment (anxiolytic and impulsivity management)	[45]
Ethanol, propylene glycol, Transcutol with a crosslinked polyacrylate polymer		1.0, 2.5, 5.0%	Alcohol-induced neurodegeneration treatment	[46]
Freeze-dried 2-hydroxyethyl cellulose/β-cyclodextrin cryogel		0.5 mg/gel	Skin cancer treatment (primary skin tumors and cutaneous metastases)	[47]
Undisclosed commercial formulation (ZYN002, Zynerba Pharmaceuticals)		250 mg/d, 500 mg/d, 750 mg/d, and 1000 mg/d 2.6 mg/kg/d, 5.3 mg/kg/d	Fragile X syndrome and 22q11.2 deletion syndrome in children Focal epilepsy in adults	[48,49,50]
Nanoemulsion (Labrasol, Tween 80, Maisine, Transcutol with isopropyl tetradecanoate and olive oil)-filled chitosan hydrogelO		1 mg/g and 10 mg/f of nanoemulsion	Epilepsy, psychiatric and skin conditions, pain, and inflammation treatment	[51]
Sodium alginate-Zn hydrogel		0.5, 1.0, 2.0, 4.0% *w*/*v*	Wound healing	[52]
Solutol HS 15, Transcutol P, isopropyl myristate, water, Sepigel 305		1% *w*/*w*	Skin diseases treatment	[53]
Propylene glycol, water hydrogel, and 3D printed Eudragit RL and tributyl citrate patch	Hydrogel Patch	1% *w*/*w*	-	[54]
Mineral oil with propylene glycol: water or propylene glycol: water: ethanol	Patch	Saturated solution	Chemotherapy side effects management (antiemetic, appetite stimulant, analgesic)	[55]
Chitosan functionalized with ZnO nanoparticles		0.5 mg/cm^3^ of ethanol	Epilepsy management	[56]
Organosilica nanoparticles with polyvinyl alcohol	Thin film	7.11 g of CBD–Silica	-	[57]
Capryol 90, Procetyl^®^ AWS, ethanol, distilled water	Microemulsion	1% *w*/*w* CBDA + THCA	-	[58]
Cholesterol, paraffin, and white Vaseline	Ointment	2 g of 20% CBD oil	Myofascial pain management	[59]
CBD seed oil		-	Skin chronic diseases (atopic dermatitis, psoriasis, infections…) Scars healing	[60]

**Table 2:** In vivo paw inflammation models for screening drugs’ anti-inflammatories properties.

Model	Advantages	Limitations
Carrageenan-Induced Edema	- Reproducible - Well-established - Bi-phasic mechanism of action	- Minimal and transient response - Solution preparation required extreme precision
Histamine/Serotonin-Induced Edema	- Suitable as a complementary method - Confirmation of biological target	- Minimal and transient response - Inappropriate for assessment of PG inhibitors
Bradykinin-Induced Edema	- Suitable as a complementary method - Confirmation of biological target	- Minimal and transient response - Inappropriate for assessment of histidine/serotonin inhibitors
Dextran-Induced Edema	- Bi-phasic mechanism of action - Good to reinforce the results of the carrageenan model	- Minimal and transient response
LPS-Induced Edema	- Suitable to assess drugs that modulate cytokine levels	- Minimal and transient response
Formalin-Induced Edema	- Biphasic behavioral response	- Pain for an extended period
CFA-Induced Edema	- Suitable to assess acute, chronic, and immune inflammation - More persistent inflammation and symptoms - Suitable to assess anti-arthritic effects	- Increase levels of stress and pain for a more extended period

## References

[1] Lodzki M., Godin B., Rakou L., Mechoulam R., Gallily R., Touitou E. (2003). Cannabidiol—Transdermal Delivery and Anti-Inflammatory Effect in a Murine Model. J. Control. Release.

[2] World Health Organization Osteoarthritis. https://www.who.int/news-room/fact-sheets/detail/osteoarthritis.

[3] Maqbool M., Fekadu G., Jiang X., Bekele F., Tolossa T., Turi E., Fetensa G., Fanta K. (2021). An up to Date on Clinical Prospects and Management of Osteoarthritis. Ann. Med. Surg..

[4] Benjamin O., Goyal A., Lappin S.L. (2023). Disease-Modifying Antirheumatic Drugs (DMARD). StatPearls [Internet].

[5] Benyamin R., Trescot A., Datta S., Buenaventura R., Adlaka R., Seghal N., Glaser S.E., Vallejo R. (2008). Opioid Complications and Side Effects. Pain Physician.

[6] Bijlsma J.W.J., Van Everdingen A.A., Jacobs J.W.G. (1995). Corticosteroids in Rheumatoid Arthritis. Clin. Immunother..

[7] Bindu S., Mazumder S., Bandyopadhyay U. (2020). Non-Steroidal Anti-Inflammatory Drugs (NSAIDs) and Organ Damage: A Current Perspective. Biochem. Pharmacol..

[8] Goodwin J.L.R., Kraemer J.J., Bajwa Z.H. (2009). The Use of Opioids in the Treatment of Osteoarthritis: When, Why, and How?. Curr. Rheumatol. Rep..

[9] Katz J.N., Arant K.R., Loeser R.F. (2021). Diagnosis and Treatment of Hip and Knee Osteoarthritis. JAMA.

[10] Mrid B.R., Bouchmaa N., Ainani H., El Fatimy R., Malka G., Mazini L. (2022). Anti-Rheumatoid Drugs Advancements: New Insights into the Molecular Treatment of Rheumatoid Arthritis. Biomed. Pharmacother..

[11] Amin M.R., Ali D.W. (2019). Pharmacology of Medical Cannabis. Adv. Exp. Med. Biol..

[12] Gülck T., Møller B.L. (2020). Phytocannabinoids: Origins and Biosynthesis. Trends Plant Sci..

[13] Pagano C., Navarra G., Coppola L., Avilia G., Bifulco M., Laezza C. (2022). Cannabinoids: Therapeutic Use in Clinical Practice. Int. J. Mol. Sci..

[14] Boyaji S., Merkow J., Elman R.N.M., Kaye A.D., Yong R.J., Urman R.D. (2020). The Role of Cannabidiol (CBD) in Chronic Pain Management: An Assessment of Current Evidence. Curr. Pain Headache Rep..

[15] Tijani A.O., Thakur D., Mishra D., Frempong D., Chukwunyere U.I., Puri A. (2021). Delivering Therapeutic Cannabinoids via Skin: Current State and Future Perspectives. J. Control. Release.

[16] Mahmoudinoodezh H., Telukutla S.R., Bhangu S.K., Bachari A., Cavalieri F., Mantri N. (2022). The Transdermal Delivery of Therapeutic Cannabinoids. Pharmaceutics.

[17] Lu H.-C., Mackie K. (2016). An Introduction to the Endogenous Cannabinoid System. Biol. Psychiatry.

[18] Almeida D.L., Devi L.A. (2020). Diversity of Molecular Targets and Signaling Pathways for CBD. Pharmacol. Res. Perspect..

[19] Howlett A.C., Abood M.E. (2017). CB 1 and CB 2 Receptor Pharmacology. Cannabinoid Pharmacol..

[20] Mlost J., Bryk M., Starowicz K. (2020). Cannabidiol for Pain Treatment: Focus on Pharmacology and Mechanism of Action. Int. J. Mol. Sci..

[21] Zou S., Kumar U. (2018). Cannabinoid Receptors and the Endocannabinoid System: Signaling and Function in the Central Nervous System. Int. J. Mol. Sci..

[22] Dos Reis Rosa Franco G., Smid S., Viegas C. (2021). Phytocannabinoids: General Aspects and Pharmacological Potential in Neurodegenerative Diseases. Curr. Neuropharmacol..

[23] Nigro E., Formato M., Crescente G., Daniele A. (2021). Cancer Initiation, Progression and Resistance: Are Phytocannabinoids from *Cannabis sativa* L. Promising Compounds?. Molecules.

[24] Borges R., Batista J., Viana R., Baetas A., Orestes E., Andrade M., Honório K., da Silva A. (2013). Understanding the Molecular Aspects of Tetrahydrocannabinol and Cannabidiol as Antioxidants. Molecules.

[25] Atalay S., Jarocka-Karpowicz I., Skrzydlewska E. (2019). Antioxidative and Anti-Inflammatory Properties of Cannabidiol. Antioxidants.

[26] Turcotte C., Blanchet M.-R., Laviolette M., Flamand N. (2016). The CB2 Receptor and Its Role as a Regulator of Inflammation. Cell. Mol. Life Sci..

[27] Ashton J., Glass M. (2007). The Cannabinoid CB2 Receptor as a Target for Inflammation-Dependent Neurodegeneration. Curr. Neuropharmacol..

[28] Pertwee R.G. (2008). The Diverse CB1and CB2receptor Pharmacology of Three Plant Cannabinoids: Δ9-Tetrahydrocannabinol, Cannabidiol and Δ9-Tetrahydrocannabivarin. Br. J. Pharmacol..

[29] Lunn C.A., Fine J.S., Rojas-Triana A., Jackson J.V., Fan X., Kung T.T., Gonsiorek W., Schwarz M.A., Lavey B., Kozlowski J.A. (2005). A Novel Cannabinoid Peripheral Cannabinoid Receptor-Selective Inverse Agonist Blocks Leukocyte Recruitment In Vivo. J. Pharmacol. Exp. Ther..

[30] Turcotte C., Chouinard F., Lefebvre J.S., Flamand N. (2015). Regulation of Inflammation by Cannabinoids, the Endocannabinoids 2-Arachidonoyl-Glycerol and Arachidonoyl-Ethanolamide, and Their Metabolites. J. Leukoc. Biol..

[31] Staton P.C., Hatcher J.P., Walker D.J., Morrison A.D., Shapland E.M., Hughes J.P., Chong E., Mander P.K., Green P.J., Billinton A. (2008). The Putative Cannabinoid Receptor GPR55 Plays a Role in Mechanical Hyperalgesia Associated with Inflammatory and Neuropathic Pain. Pain.

[32] Etemad L., Karimi G., Alavi M.S., Roohbakhsh A. (2022). Pharmacological Effects of Cannabidiol by Transient Receptor Potential Channels. Life Sci..

[33] Muller C., Morales P., Reggio P.H. (2019). Cannabinoid Ligands Targeting TRP Channels. Front. Mol. Neurosci..

[34] Xu H., You M., Shi H., Hou Y. (2014). Ubiquitin-Mediated NFκB Degradation Pathway. Cell. Mol. Immunol..

[35] Health Canada Review of Cannabidiol: Report of the Science Advisory Committee on Health Products Containing Cannabis. Canada. 28 July 2022. https://www.canada.ca/en/health-canada/corporate/about-health-canada/public-engagement/external-advisory-bodies/health-products-containing-cannabis/review-cannabidiol-health-products-containing-cannabis.html.

[36] Barnes M.P. (2006). Sativex^®^: Clinical Efficacy and Tolerability in the Treatment of Symptoms of Multiple Sclerosis and Neuropathic Pain. Expert Opin. Pharmacother..

[37] Office of the Commissioner (2018). FDA Approves First Drug Comprised of an Active Ingredient Derived from Marijuana to Treat Rare, Severe Forms of Epilepsy.

[38] Office of the Commissioner (2024). FDA Regulation of Cannabis and Cannabis-Derived Products: Q&A.

[39] O’Donnell B., Meissner H., Gupta V. (2023). Dronabinol. StatPearls [Internet].

[40] Throckmorton D. (2021). FDA Role in Regulation of Cannabis Products. https://www.fda.gov/media/152407/download.

[41] (2014). Orphan Designation for Treatment of Dravet Syndrome.

[42] European Medicines Agency (2022). European Medicines Agency Decision: P/0110/2022.

[43] Kováčik A., Kopečná M., Vávrová K. (2020). Permeation Enhancers in Transdermal Drug Delivery: Benefits and Limitations. Expert Opin. Drug Deliv..

[44] Hammell D.C., Zhang L.P., Ma F., Abshire S.M., McIlwrath S.L., Stinchcomb A.L., Westlund K.N. (2015). Transdermal Cannabidiol Reduces Inflammation and Pain-Related Behaviours in a Rat Model of Arthritis. Eur. J. Pain.

[45] Gonzalez-Cuevas G., Martin-Fardon R., Kerr T.M., Stouffer D.G., Parsons L.H., Hammell D.C., Banks S.L., Stinchcomb A.L., Weiss F. (2018). Unique Treatment Potential of Cannabidiol for the Prevention of Relapse to Drug Use: Preclinical Proof of Principle. Neuropsychopharmacology.

[46] Liput D.J., Hammell D.C., Stinchcomb A.L., Nixon K. (2013). Transdermal Delivery of Cannabidiol Attenuates Binge Alcohol-Induced Neurodegeneration in a Rodent Model of an Alcohol Use Disorder. Pharmacol. Biochem. Behav..

[47] Momekova D., Danov Y., Momekov G., Ivanov E., Petrov P. (2021). Polysaccharide Cryogels Containing β-Cyclodextrin for the Delivery of Cannabidiol. Pharmaceutics.

[48] Scheffer I.E., Hulihan J., Messenheimer J., Ali S., Keenan N., Griesser J., Gutterman D.L., Sebree T., Sadleir L.G. (2021). Safety and Tolerability of Transdermal Cannabidiol Gel in Children with Developmental and Epileptic Encephalopathies: A Nonrandomized Controlled Trial. JAMA Netw. Open.

[49] O’Brien T.J., Berkovic S.F., French J.A., Messenheimer J.A., Sebree T.B., Bonn-Miller M.O., Gutterman D.L., STAR 1/STAR 2 Study Group (2022). Adjunctive Transdermal Cannabidiol for Adults with Focal Epilepsy: A Randomized Clinical Trial. JAMA Netw. Open.

[50] Berry-Kravis E., Hagerman R., Budimirovic D., Erickson C., Heussler H., Tartaglia N., Cohen J., Tassone F., Dobbins T., Merikle E. (2022). A Randomized, Controlled Trial of ZYN002 Cannabidiol Transdermal Gel in Children and Adolescents with Fragile X Syndrome (CONNECT-FX). J. Neurodev. Disord..

[51] Demisli S., Galani E., Goulielmaki M., Kyrilis F.L., Ilić T., Hamdi F., Crevar M., Kastritis P.L., Pletsa V., Nallet F. (2023). Encapsulation of Cannabidiol in Oil-In-Water Nanoemulsions and Nanoemulsion-Filled Hydrogels: A Structure and Biological Assessment Study. J. Colloid Interface Sci..

[52] Zheng Z., Qi J., Hu L., Ouyang D., Wang H., Sun Q., Lin L., You L., Tang B. (2021). A Cannabidiol-Containing Alginate Based Hydrogel as Novel Multifunctional Wound Dressing for Promoting Wound Healing. Mater. Sci. Eng. C.

[53] Vanti G., Grifoni L., Bergonzi M.C., Antiga E., Montefusco F., Caproni M., Bilia A.R. (2021). Development and Optimisation of Biopharmaceutical Properties of a New Microemulgel of Cannabidiol for Locally-Acting Dermatological Delivery. Int. J. Pharm..

[54] Casiraghi A., Musazzi U.M., Centin G., Franzè S., Minghetti P. (2020). Topical Administration of Cannabidiol: Influence of Vehicle-Related Aspects on Skin Permeation Process. Pharmaceuticals.

[55] Stinchcomb A.L., Valiveti S., Hammell D.C., Ramsey D.R. (2004). Human Skin Permeation of Δ8-Tetrahydrocannabinol, Cannabidiol and Cannabinol. J. Pharm. Pharmacol..

[56] Radwan-Pragłowska J., Janus Ł., Piątkowski M., Sierakowska A., Szajna E., Matýsek D., Bogdał D. (2021). Development of Stimuli-Responsive Chitosan/ZnO NPs Transdermal Systems for Controlled Cannabidiol Delivery. Polymers.

[57] Khabir Z., Partalis C., Panchal J.V., Deva A., Khatri A., Garcia-Bennett A. (2023). Enhanced Skin Penetration of Cannabidiol Using Organosilane Particles as Transdermal Delivery Vehicles. Pharmaceutics.

[58] Park C., Zuo J., Somayaji V., Lee B.-J., Löbenberg R. (2021). Development of a Novel Cannabinoid-Loaded Microemulsion towards an Improved Stability and Transdermal Delivery. Int. J. Pharm..

[59] Nitecka-Buchta A., Nowak-Wachol A., Wachol K., Walczyńska-Dragon K., Olczyk P., Batoryna O., Kempa W., Baron S. (2019). Myorelaxant Effect of Transdermal Cannabidiol Application in Patients with TMD: A Randomized, Double-Blind Trial. J. Clin. Med..

[60] Palmieri B., Laurino C., Vadalà M. (2019). A Therapeutic Effect of Cbd-Enriched Ointment in Inflammatory Skin Diseases and Cutaneous Scars Clinical Trial. Clin. Ter..

[61] Emanet M., Ciofani G. (2023). Ethosomes as Promising Transdermal Delivery Systems of Natural-Derived Active Compounds. Adv. NanoBiomed Res..

[62] Ethier A., Bansal P., Baxter J., Langley N., Richardson N., Patel A.M., Langley N., Michniak-Kohn B., Osborne D.W. (2019). The Role of Excipients in the Microstructure of Topical Semisolid Drug Products. The Role of Microstructure in Topical Drug Product Development.

[63] Javadzadeh Y., Adibkia K., Hamishekar H., Dragicevic N., Maibach H.I. (2015). Transcutol^®^ (Diethylene Glycol Monoethyl Ether): A Potential Penetration Enhancer. Percutaneous Penetration Enhancers Chemical Methods in Penetration Enhancement.

[64] Cova T.F., Murtinho D., Pais A.A.C.C., Valente A.J.M. (2018). Combining Cellulose and Cyclodextrins: Fascinating Designs for Materials and Pharmaceutics. Front. Chem..

[65] Taokaew S., Kaewkong W., Kriangkrai W. (2023). Recent Development of Functional Chitosan-Based Hydrogels for Pharmaceutical and Biomedical Applications. Gels.

[66] Tomić S.L., Babić Radić M.M., Vuković J.S., Filipović V.V., Nikodinovic-Runic J., Vukomanović M. (2023). Alginate-Based Hydrogels and Scaffolds for Biomedical Applications. Mar. Drugs.

[67] Berenguer D., Sosa L., Alcover M., Sessa M., Halbaut L., Guillén C., Fisa R., Calpena-Campmany A.C., Riera C. (2019). Development and Characterization of a Semi-Solid Dosage Form of Meglumine Antimoniate for Topical Treatment of Cutaneous Leishmaniasis. Pharmaceutics.

[68] Morais R.P., Hochheim S., de Oliveira C.C., Riegel-Vidotti I.C., Marino C.E.B. (2022). Skin Interaction, Permeation, and Toxicity of Silica Nanoparticles: Challenges and Recent Therapeutic and Cosmetic Advances. Int. J. Pharm..

[69] Patil K.R., Mahajan U.B., Unger B.S., Goyal S.N., Belemkar S., Surana S.J., Ojha S., Patil C.R. (2019). Animal Models of Inflammation for Screening of Anti-Inflammatory Drugs: Implications for the Discovery and Development of Phytopharmaceuticals. Int. J. Mol. Sci..

[70] Ghorbanzadeh B., Mansouri M., Hemmati A., Naghizadeh B., Mard S., Rezaie A. (2015). A Study of the Mechanisms Underlying the Anti-Inflammatory Effect of Ellagic Acid in Carrageenan-Induced Paw Edema in Rats. Indian J. Pharmacol..

[71] Fehrenbacher J.C., Vasko M.R., Duarte D.B. (2012). Models of Inflammation: Carrageenan- or Complete Freund’s Adjuvant-Induced Edema and Hypersensitivity in the Rat. Curr. Protoc. Pharmacol..

[72] Tamaddonfard E., Farshid A.A., Hosseini L. (2012). Crocin Alleviates the Local Paw Edema Induced by Histamine in Rats. Avicenna J. Phytomed..

[73] Akpinar E. (2021). Experimental Inflammation Models Created in Laboratory Animals. Atatürk Univ. J. Vet. Sci..

[74] Rex D.A.B., Deepak K., Vaid N., Dagamajalu S., Kandasamy R.K., Flo T.H., Keshava Prasad T.S. (2021). A Modular Map of Bradykinin-Mediated Inflammatory Signaling Network. J. Cell Commun. Signal..

[75] Coura C.O., Souza R.B., Rodrigues J.A.G., de Sousa Oliveira Vanderlei E., de Araújo I.W.F., Ribeiro N.A., Frota A.F., Ribeiro K.A., Chaves H.V., Pereira K.M.A. (2015). Mechanisms Involved in the Anti-Inflammatory Action of a Polysulfated Fraction from *Gracilaria cornea* in Rats. PLoS ONE.

[76] Xu M., Chen Q., Fan R., Wang J., Li Y. (2019). Anti-Inflammation Effect of Small Molecule Oligopeptides Prepared from *Panax ginseng* C. A. Meyer in Rats. Molecules.

[77] Vajja B.N.L., Juluri S., Kumari M., Kole L., Chakrabarti R., Joshi V.D. (2004). Lipopolysaccharide-Induced Paw Edema Model for Detection of Cytokine Modulating Anti-Inflammatory Agents. Int. Immunopharmacol..

[78] Damas J., Liégeois J.F. (1999). The Inflammatory Reaction Induced by Formalin in the Rat Paw. Naunyn-Schmiedeberg’s Arch. Pharmacol..

